# lncRNA RUNDC3A-AS1 Regulates Proliferation and Apoptosis of Thyroid Cancer Cells via the miR-151b/SNRPB Axis

**DOI:** 10.1155/2022/9433434

**Published:** 2022-02-22

**Authors:** Yan Deng, Jie Wu, Xin Li

**Affiliations:** ^1^Department of Nuclear Medicine, Wuhan Fifth Hospital, Wuhan 430050, Hubei, China; ^2^Department of Laboratory, Hubei No. 3 People's Hospital of Jianghan University, Wuhan 430033, Hubei, China

## Abstract

The number of thyroid cancer (THCA) cases has increased dramatically worldwide. Many previous reports have confirmed that lncRNA is involved in the pathogenesis of THCA. However, the role and mechanism of lncRNA RUNDC3A-AS1 in THCA have not been studied. We intended to explore the effect of RUNDC3A-AS1 on the proliferation and apoptosis of THCA cells. Relative expression levels of RUNDC3A-AS1, microRNA (miR)-151b, and small nuclear ribonucleoprotein polypeptides B and B1 (SNRPB) were examined by reverse transcription quantitative polymerase chain reaction (RT-qPCR) in THCA cells. The localization of RUNDC3A-AS1 in THCA cells was detected by subcellular fractionation assay. The cell proliferation was tested by 5-ethynyl-2′-deoxyuridine (EdU), cell counting kit-8 (CCK-8), and 3-(4,5-dimethylthiazol-2-yl)-2,5-diphenyltetrazolium bromide (MTT) assays. Flow cytometry was used to examine the cell apoptosis capacity. The relationships between RUNDC3A-AS1 and miR-151b or miR-151b and SNRPB were verified by luciferase reporter assay. The protein level was detected by Western blot analysis. RUNDC3A-AS1 exhibited high expression in THCA cells. RUNDC3A-AS1 knockdown suppressed cell proliferation but induced cell apoptosis. Importantly, RUNDC3A-AS1 targeted miR-151b to regulate the SNRPB expression. In rescue assays, SNRPB overexpression partially reversed the suppressive effect of RUNDC3A-AS1 knockdown on cell proliferation and the promotive effect of RUNDC3A-AS1 knockdown on cell apoptosis. The RUNDC3A-AS1/miR-151b/SNRPB axis regulated THCA cell proliferation and apoptosis, which provides novel insight into THCA investigation at the molecular level.

## 1. Introduction

As a common malignant tumor, thyroid cancer (THCA) has the highest incidence rate in the world [1–3]. THCA accounts for 13% of all invasive tumors in adolescents and young adults. In women, it is three times more common [4, 5]. The treatment of advanced and metastatic THCA is still very challenging. However, significant advances have been made in the unique molecular characteristics of THCA [6]. Therefore, it is imperative for exploration and understanding of the molecular mechanisms underlying THCA.

Long noncoding RNAs (lncRNAs) are noncoding RNAs whose length is greater than 200 nt and which do not have protein-coding ability. New evidence suggests that lncRNAs are involved in the oncogenesis of many malignancies [7, 8]. For example, the lncRNA CDC6 acts as an oncogene in breast cancer [9]. In epithelial ovarian cancer, lncRNA TUG1 promotes cell proliferation and suppresses cell apoptosis by regulating AURKA [10]. lncRNA-RMRP affects bladder cancer through miR-206 [11]. Additionally, numerous lncRNAs like XIST, SPRY4-IT, and PTCSC2 have been demonstrated to facilitate tumorigenesis and progression of THCA [12–14]. It has been identified that RUNDC3A-AS1 expression was notably associated with THCA, suggesting that lncRNA RUNDC3A-AS1 could be a useful prediction factor for the survival of THCA patients [15, 16]. However, the function and mechanism of RUNDC3A-AS1 in THCA have not been studied yet.

MicroRNAs are endogenous short noncoding single-stranded RNAs that can induce messenger RNA (mRNA) to block translation [17, 18]. Accumulating studies have reported the potential of miRNAs in the process of THCA. For instance, downregulation of miR-17-5p affects the development of THCA by targeting PTEN [19]. The introduction of miR-17-5p inhibited cell viability, migration, and invasion in THCA cells [20]. miR-205 mediates cell migration and proliferation of THCA cells by targeting CCNB2 [21]. We attempted to identify miRNAs that may interact with RUNDC3A-AS1. Herein, we used online tools to determine downstream target miRNA of RUNDC3A-AS1 and miR-151b was confirmed for further investigation.

According to the abovementioned research, this study preliminarily explored the functions of lncRNA RUNDC3A-AS1 in THCA cells and determined the molecular mechanisms involved. Thus, we aim to provide a novel argument for exploring the role of RUNDC3A-AS1 in the development and treatment of THCA.

## 2. Materials and Methods

### 2.1. Cell Culture

One normal thyroid epithelial cell line (Nthy-ori 3-1) and three papillary thyroid carcinoma cell lines (K1, TPC-1, and IHH4) were purchased from the American Type Culture Collection (Rockville, MD, USA). All cell lines were preserved in DMEM (GIBCO-BRL) medium, which contained 10% FBS, 100 mg/ml streptomycin, and 100 U/ml penicillin. The cells were stored at 37°C in a humidified atmosphere of 5% CO_2_.

### 2.2. Cell Transfection

The small interference RNA (si-RUNDC3A-AS1#1 or #2), miR-151b mimics, and corresponding negative controls (NCs) were made by GenePharma (Shanghai, China). Full-length sequences of SNRPB were synthesized and subcloned into the pcDNA3.1 (Sangon, Shanghai, China) plasmid to produce pcDNA3.1/SNRPB. Lipofectamine 2000 (Invitrogen) was used to conduct all transfections. After 48 h, the cells were harvested. RT-qPCR was used to measure the efficiency of the transfection.

### 2.3. Reverse Transcription-Quantitative Polymerase Chain Reaction (RT-qPCR)

Total RNA was isolated from THCA cells using TRIzol reagent (Invitrogen). The cDNA was synthesized using the PrimeScript RT reagent Master Mix (Takara). RT‐qPCR was performed by the SYBR Premix Ex Taq kit (Takara). Glyceraldehyde‐3‐phosphate dehydrogenase (GAPDH) or U6 small nuclear RNA (snRNA) was used as the control. The PCR primer sequences were shown as followings: GAPDH, forward, 5′-GCA CCG TCA AGG CTG AGA AC-3′ and reverse, 5′-TGG TGA AGA CGC CAG TGG A-3′; U6, forward, 5′ATA CAG AGA AAG TTA GCA CGG-3′and reverse, 5′-GGA ATG CTT CAA AGA GTT GTG-3′; RUNDC3A-AS1, forward, 5′-TCC AGA ACT GGA AAC TAC CC-3′ and reverse, 5′- GCC ATT TGT CAT TGT CTT CCT-3′; si-RUNDC3A-AS1#1, forward, 5′-AAU CUG AAU CAA UGU AGA GAC-3′ and reverse, 5′- CUC UAC AUU GAU UCA GAU UUG -3′; miR-151b, forward, 5′- ACA CTC CAG CTG GGT CGA GGA GCT CA′ and reverse, 5′- TGG TGT CGT GGA GTC G-3′; miR-151b mimics, 5′-UCG AGG AGC UCA CAG UCU-3′; SNRPB, forward, 5′-CCG GAT CTT CAT TGG CAC CT-3′ and reverse, 5′-AGG ACT CGC TTC TCT TCC CT-3′. The relative gene expression was normalized to control by the 2^−ΔΔCt^ method.

### 2.4. Subcellular Fractionation Assay

NE-PER Nuclear and Cytoplasmic Extraction Reagents (Thermo Scientific, Waltham, MA, USA) were used to extract the cytoplasm and nuclear extracts of THCA cells. To identify the levels of nuclear control (U6), cytoplasmic control (GAPDH), and lncRNA RUNDC3A-AS1, RT-qPCR was used to analyze the RNAs isolated from the nucleus or cytoplasm.

### 2.5. Cell Counting Kit-8 (CCK-8) Assay

THCA cells (1 × 10^4^ cells/well) were transfected and then seeded in 96-well plates. At different time points (24, 48, and 72 h), CCK-8 solution (Dojindo Molecular Technologies, Inc.) (10 *µ*l) was added to each well after the 48 h culture. Then, cells were cultured for an additional 2 h at 37°C. Viable cells were detected by absorbance at a 450 nm wavelength with a microplate reader (Bio-Rad, Hercules, CA, USA).

### 2.6. 5-Ethynyl-2′-Deoxyuridine (EdU) Assay

The proliferation ability of transfected cells was evaluated using the EdU cell proliferation kit (RiboBio, Guangzhou, China). In brief, cells in the growth phase were treated with EdU for 2 h. After washing the cells with 0.5 g/ml of PBS three times, the nuclei were counterstained with DAPI (Invitrogen) at room temperature and darkness for 10 min. DAPI-labeled cells were washed three times with PBS. A fluorescence microscope (Carl Zeiss, Oberkochen, Germany) was used to visualize the results. All samples were assayed three times.

### 2.7. Flow Cytometry

For the cell apoptosis assay, cells (1 × 10^6^ cells/mL) were treated with 1 × binding buffer and double stained with propidium iodide (KeyGen, Nanjing, China). Cell apoptosis was detected by FlowJo software (Tree Star Corp, Ashland, OR) and FACSCalibur (BD Biosciences).

### 2.8. Western Blot Analysis

After the isolated proteins were transferred to polyvinylidene difluoride (PVDF) membranes (Beyotime), the membranes were placed in Tris-buffered saline with Tween buffer and blocked with 5% skim milk. Primary antibodies were then used to incubate the membranes. Primary antibodies include anti‐cleaved caspase 3 (ab32042, 1 : 500, Abcam), anti‐Bax (ab32503, 1 : 1000, Abcam), anti‐Bcl-2 (ab32124, 1 : 1000, Abcam), anti‐SNRPB (ab155026, 1 : 500, Abcam), and anti‐GAPDH (ab8245, 1 : 500, Abcam). Next, the membranes were incubated with the secondary antibody. Finally, the immunoblots were visualized by ImageJ software (version 1.6.0, Windows, NIH).

### 2.9. Luciferase Reporter Assay

To construct the luciferase reporters, the sequences of wild‐type RUNDC3A-AS1 (RUNDC3A-AS1‐WT), mutant RUNDC3A-AS1 (RUNDC3A-AS1‐MUT), wild‐type 3′ untranslated regions (UTR) of SNRPB (SNRPB 3′UTR‐WT), and mutant 3′UTR of SNRPB (SNRPB 3′UTR‐MUT) were inserted into the pmirGLO luciferase vectors (GeneCreate, Wuhan, China). Then, THCA cells were cotransfected with NC mimics or miR‐151b using Lipofectamine 2000 (Invitrogen). Luciferase activity was examined by the luciferase reporter assay kit (Promega, Madison, WI, USA).

### 2.10. Bioinformatics Analysis

The expression levels of RUNDC3A-AS1, miR‐151b, and SNRPB in 510 cancer and 58 normal samples in thyroid carcinoma were obtained from the online starBase v2.0 database (http://starbase.sysu.edu.cn). The target predictions between RUNDC3A-AS1/miR‐151b were also analyzed using the starBase. The binding sites between RUNDC3A-AS1 and miR‐151b or miR‐151b and SNRPB were predicted using the TargetScan database (https://www.targetscan.org).

### 2.11. Statistical Analysis

All results were recorded as mean ± standard deviation (SD) and analyzed by SPSS 21.0 software (IBM Corp. Armonk, NY, USA). *T*-tests were used to compare between two groups. The significance of differences among multiple groups was estimated by one-way ANOVA. *P* < 0.05 were considered significant.

## 3. Results

### 3.1. RUNDC3A-AS1 Is Highly Expressed in THCA

**Figure 1:**
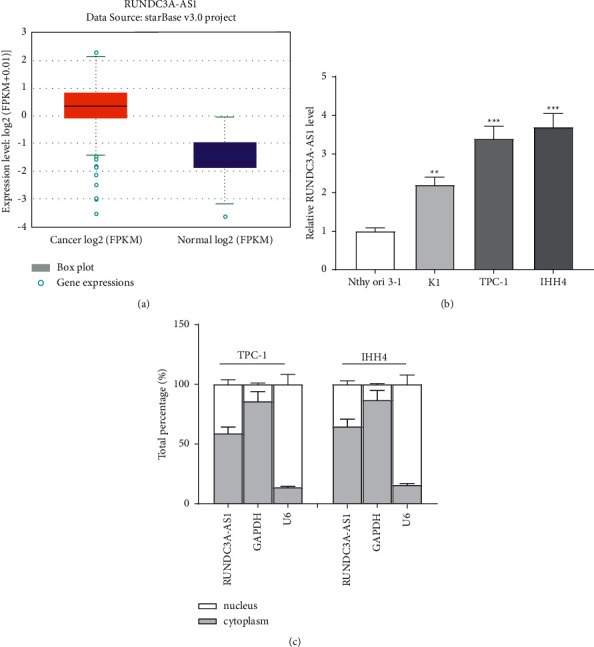
RUNDC3A-AS1 is highly expressed in THCA. (a) ENCORI showed the high expression of RUNDC3A-AS1 in THCA. (b) Expression of RUNDC3A-AS1 in K1, TPC-1, and IHH4 cell lines was examined by RT-qPCR. (c) The cytoplasmic and nuclear levels of RUNDC3A-AS1 in TPC-1 and IHH4 cells were analyzed by a subcellular fractionation assay. ^*∗∗*^*P* < 0.01; ^*∗∗∗*^*P* < 0.001.

According to the ENCORI website, RUNDC3A-AS1 showed high expression in 510 THCA samples, compared with 58 normal samples (Figure 1(a)). RT-qPCR showed that the RUNDC3A-AS1 expression in the THCA cell lines was significantly increased, compared with the Nthy-ori3-1 cell line. Among all the THCA cell lines, TPC-1 and IHH4 exhibited the highest expression of RUNDC3A-AS1 (Figure 1(b)). Therefore, the THCA cells TPC-1 and IHH4 were selected for the subsequent experiments. Furthermore, the subcellular fractionation assay unraveled that RUNDC3A-AS1 was primarily located in the cytoplasm (Figure 1(c)). These results suggested that RUNDC3A-AS1 showed high expression in THCA cells and may play a role at the post-transcriptional level.

### 3.2. Interfering RUNDC3A-AS1 Inhibits Cell Proliferation and Promotes Cell Apoptosis

**Figure 2:**
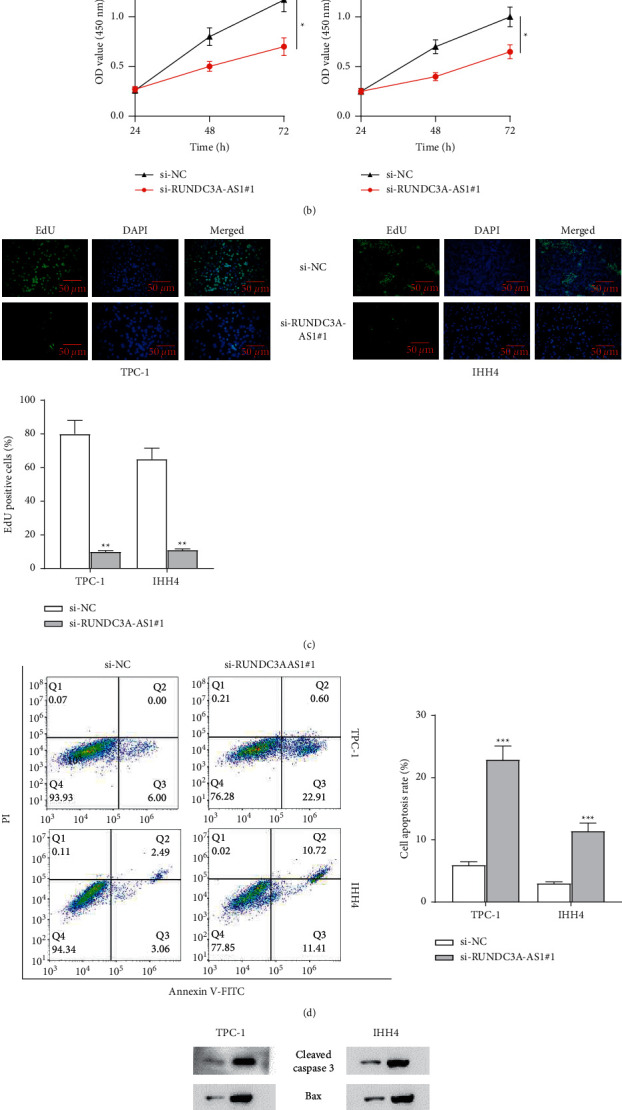
Interfering RUNDC3A-AS1 inhibits cell proliferation and promotes cell apoptosis. (a) The knockdown efficiency of RUNDC3A-AS1 was examined by RT-qPCR. (b) Cell proliferation was examined by the CCK-8 assay. (c) The EdU assay was conducted to detect cell proliferation. (d) THCA cell apoptosis was examined by flow cytometry. (e) Apoptosis-related protein level was examined by western blot analysis. ^*∗∗*^*P* < 0.01; ^*∗∗∗*^*P* < 0.001.

The RUNDC3A-AS1 siRNAs were transfected into the cells (TPC-1 and IHH4) to evaluate the effect of RUNDC3A-AS1 knockdown on cell proliferation and apoptosis. RT-qPCR showed that the expression level of RUNDC3A-AS1 was significantly decreased in the cells transfected with both si-RUNDC3A-AS1#1 and si-RUNDC3A-AS1#2. However, RUNDC3A-AS1 expression was lower in the cells transfected with si-RUNDC3A-AS1#1 than si-RUNDC3A-AS1#2. Thus, we used si-RUNDC3A-AS1#1 for silencing RUNDC3A-AS1 in subsequent experiments (Figure 2(a)). According to the results of the CCK-8 assay, RUNDC3A-AS1 knockdown significantly suppressed the THCA cell viability (Figure 2(b)). Similarly, the EdU assay also showed decreased cell proliferation after silencing RUNDC3A-AS1 (Figure 2(c)). Compared with the control cells, THCA cells with RUNDC3A-AS1 knockdown significantly increased apoptosis rate according to the flow cytometry (Figure 2(d)). Furthermore, knockdown of RUNDC3A-AS1 significantly decreased the protein expression of the apoptosis suppressor gene Bcl-2, while increasing the apoptosis-promoting genes cleaved-caspase-3 and Bax (Figure 2(e)). Thus, we recognized that RUNDC3A-AS1 knockdown inhibited cell proliferation, whereas promoted cell apoptosis in THCA cell lines.

### 3.3. RUNDC3A-AS1 Binds to miR-151b

**Figure 3:**
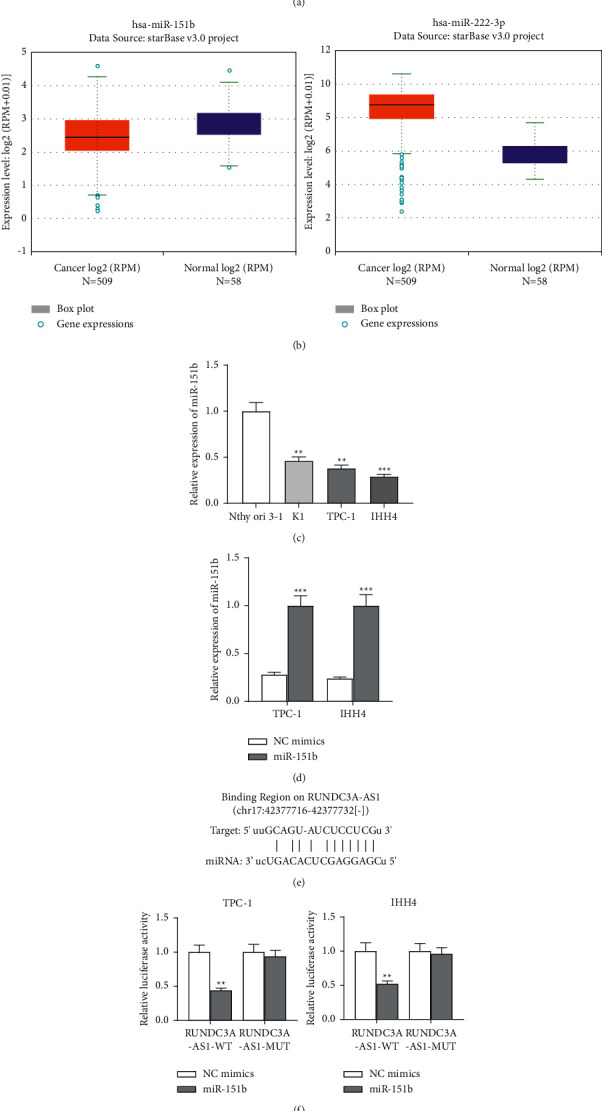
RUNDC3A-AS1 binds to miR-151b. (a) ENCORI showed two miRNAs that have binding sites with RUNDC3A-AS1 (CLIP data ≥ 1, Pan-cancer ≥ 10). (b) ENCORI showed the low expression of miR-151b in THCA. (c) The relative expression level of miR-151b in THCA cell lines was examined by RT-qPCR. (d) Overexpression efficiency of miR-151b was examined by RT-qPCR. (e) TargetScan predicted the binding sites between RUNDC3A-AS1 and miR-151b. (f) Luciferase reporter gene assay showed that miR-151b decreased the luciferase activity in the RUNDC3A-AS1-WT group. ^*∗∗*^*P* < 0.01; ^*∗∗∗*^*P* < 0.001.

The ENCORI website screened out two miRNAs, miR-151b and miR-222-3p, which have binding sites with RUNDC3A-AS1 (Figure 3(a)). The website continued to show the low expression of miR-151b but high expression of miR-222-3p in THCA samples, compared with the normal samples (Figure 3(b)). Thus, we chose miR-151b for further investigation. Then, we confirmed that miR-151b expression was decreased in THCA cell lines, compared with the control group (Figure 3(c)). RT-qPCR showed a significant high expression level of miR-151b in the cells transfected with miR-151b mimics, compared with that in the control group (Figure 3(d)). TargetScan predicted the binding sites between miR-151b and RUNDC3A-AS1 (Figure 3(e)). Then, we found that miR-151b inhibited the luciferase activity of RUNDC3A-AS1 WT-3′UTR but had no significant effect on the RUNDC3A-AS1 MUT-3′UTR (Figure 3(f)). Together, these data suggested that RUNDC3A acted as a ceRNA for miR-151b.

### 3.4. SNRPB Is a Target Gene of miR-151b

**Figure 4:**
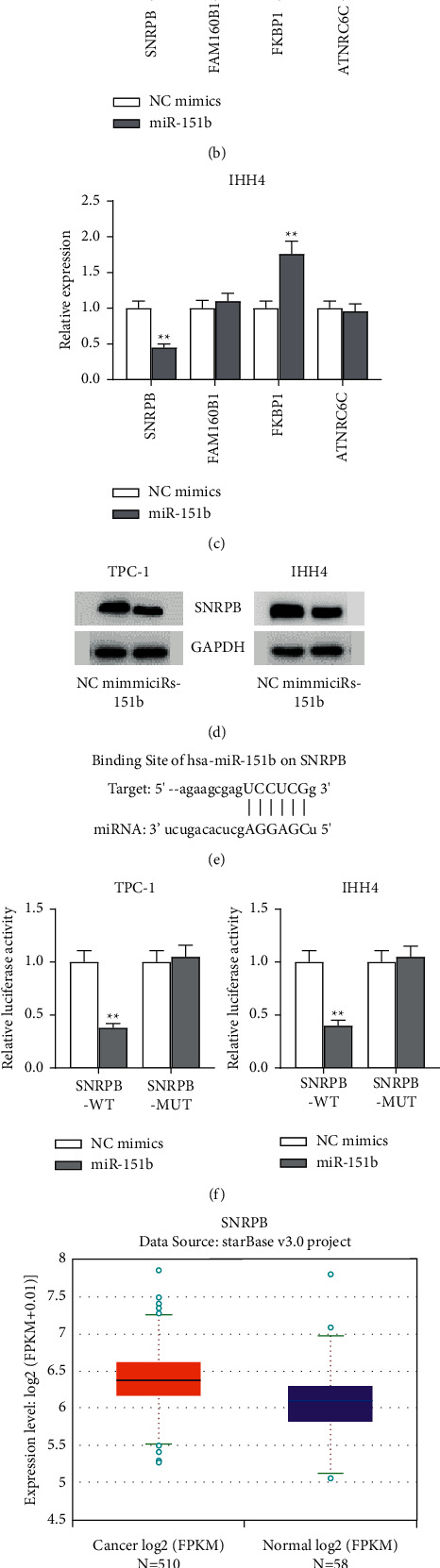
SNRPB is a target gene of miR-151b. (a) ENCORI predicted the target genes of miR-151b. (b, c) SNRPB showed the lowest expression level after miR-151b overexpression in TPC-1 and IHH4 cells. (d) The protein level of SNRPB was detected by western blot. (e) TargetScan predicted the binding site between miR-151b and SNRPB. (f) Luciferase reporter gene assay showed that miR-151b decreased the luciferase activity in the SNRPB-WT group. (g) ENCORI showed the high expression of SNRPB in THCA samples. (h) The expression level of SNRPB in THCA cell lines was examined by RT-qPCR. ^*∗*^*P* < 0.05; ^*∗∗*^*P* < 0.01.

ENCORI predicted target genes, and CLIP data ranked the following top four: SNRPB, FAM160B1, FKBP1 and TNRC6C (Figure 4(a)). When we transfected target genes with miR-151b mimics, SNRPB showed the lowest expression in both TPC-1 and IHH4 cells (Figures 4(b)–4(c)). The western blot showed a decreased protein level of SNRPB after miR-151b overexpression (Figure 4(d)). TargetScan predicted the binding sites between miR-151b and SNRPB (Figure 4(e)). To determine whether miR-151b directly interacted with SNRPB in THCA cells, luciferase reporter assay was performed (Figure 4(f)). Compared with NC mimics, cotransfection of the wild-type SNRPB vector (SNRPB-WT) with miR-151b mimics significantly reduced luciferase activity, while there was no change in mutant SNRPB vector (SNRPB-MUT) activity. In addition, according to the ENCORI website, SNRPB shows a high expression level in 510 cancer samples, compared with the 58 normal samples in THCA. (Figure 4(g)). Similarly, SNRPB showed a higher expression level in THCA cells than in Nthy-ori 3-1 (Figure 4(h)). All these results indicated that SNRPB is bound to miR-151b.

### 3.5. Overexpression of SNRPB Reverses the Effects of RUNDC3A-AS1 Knockdown on Cell Proliferation and Apoptosis

**Figure 5:**
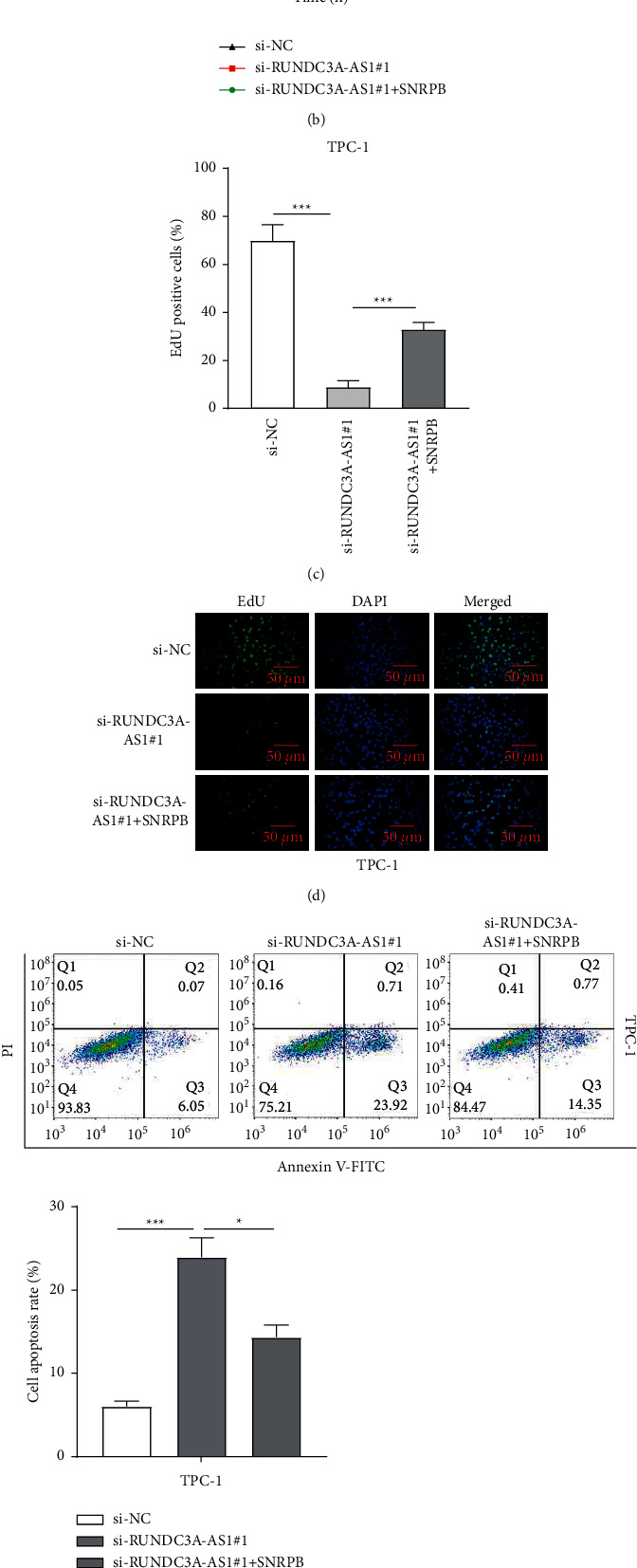
Overexpression of SNRPB reverses the effects of RUNDC3A-AS1 knockdown on cell proliferation and apoptosis. (a) RT-qPCR showed that SNRPB overexpression reversed the expression level of SNRPB caused by RUNDC3A-AS1 knockdown. (b) Cell viability was examined by the CCK-8 assay. (c, d) The EdU assay was conducted to detect cell proliferation. (e) Flow cytometry was used to test cell apoptosis. (f) The Western blot was employed to examine the protein level of apoptosis-related factors. ^*∗*^*P* < 0.05; ^*∗∗∗*^*P* < 0.001.

According to RT-qPCR analysis, RUNDC3A-AS1#1 knockdown decreased the expression level of SNRPB, whereas RUNDC3A-AS1#1 knockdown with SNRPB overexpression reversed the reduction. Consistently, the protein level of SNRPB was suppressed by si-RUNDC3A-AS1#1 but promoted by si-RUNDC3A-AS1#1+ pcDNA3.1-SNRPB (Figure 5(a)). According to the CCK-8 and EdU assays, si-RUNDC3A-AS1#1 reduced the cell proliferation, whereas SNRPB overexpression reversed the reduction and increased the cell proliferation in TPC-1 cells (Figures 5(b)–5(d)). Flow cytometry was used to detect the cell apoptosis, which showed high apoptosis rate in the si-RUNDC3A-AS1#1 group, but a lower rate in the si-RUNDC3A-AS1#1+ pcDNA3.1-SNRPB group (Figure 5(e)). Mechanically, SNRPB reversed the increased protein level of cleaved caspase 3 and Bax and decreased protein level of Bcl-2 (Figure 5(f)). These results revealed that overexpression of SNRPB reversed the effects of RUNDC3A-AS1 knockdown on THCA cell proliferation and apoptosis.

## 4. Discussion

THCA has a high incidence in China [22]. Numerous lncRNAs have been documented to contribute to many biological functions including tumorigenesis [23–25]. Several of them have been reported to affect the development of THCA. For example, lncRNA LINC00514 knockdown directs the inhibitory effect on the malignant behaviors of papillary THCA through the miR-204-3p/CDC23 axis [26]. lncRNA NEAT1 acts as an oncogene in papillary thyroid cancer by regulating the miR‐129‐5p/KLK7 axis [27]. Furthermore, the lncRNA LINC00673 promotes proliferation, metastasis, and epithelial-mesenchymal transition in THCA [28]. Although it has been reported that the RUNDC3A-AS1 level may relate to the survival of THCA patients, the concrete role and mechanism of RUNDC3A-AS1 in THCA development has not been investigated. Bioinformatic analysis shows that RUNDC3A-AS1 is overexpressed in THCA samples. In our study, we analyzed RUNDC3A-AS1 expression in THCA cell lines by RT-qPCR and found that RUNDC3A-AS1 was significantly upregulated in THCA cells, and the knockdown of RUNDC3A-AS1 inhibited THCA cell proliferation and promoted cell apoptosis. These results confirmed the role of RUNDC3A-AS1 as an oncogene in THCA.

lncRNAs interact with miRNAs by acting as competitive endogenous RNAs (ceRNAs) to regulate tumor cells [29]. For example, lncRNA HOXA11-AS targets miR-124 to promote proliferation and invasion in human nonsmall cell lung cancer cells [30]. LINC00520 promotes the progression of colorectal cancer by serving as a ceRNA for miR-577 [31]. We found that miR-151b has a binding site for RUNDC3A-AS1 through online tools. In previous studies, miR-151b was shown to be downregulated in intramucosal gastric cancer [32]. In addition, miR-151b also showed aberrant expression in osteosarcoma specimens [33]. However, the role of miR-151b in THCA has not been studied. In this study, miR-151b was downregulated in THCA cell lines. According to the results of the ENCORI website and luciferase reporter assay, we primarily demonstrated that miR‐151b was bound to RUNDC3A-AS1, suggesting that RUNDC3A-AS1 targeted miR-151b to regulate the proliferation and apoptosis of THCA cells.

It is known that the small nuclear ribonucleoprotein polypeptides B and B1 (SNRPB) genes play a significant role in regulating the pre-mRNA [34]. Presently, SNRPB was reported to modulate cervical cancer cell proliferation, migration, invasion, and apoptosis [35]. It is also reported that SNRPB facilitates nosmall-cell lung cancer tumorigenesis via regulation of RAB26 expression [36]. In addition, SNRPB promotes glycolysis in hepatocellular carcinoma cells [37]. However, the role of SNRPB in THCA is still unclear. In our study, SNRPB showed a significantly high expression level in THCA and directly interacted with miR-151b, so we chose it for the rescue assay. Notably, we found that overexpression of SNRPB reversed the inhibitory effect of RUNDC3A-AS1 knockdown on cell proliferation and the promotive effect of RUNDC3A-AS1 knockdown on cell apoptosis. However, there is still some deficiency in this study, such as lacking in *vivo* experiments to validate our results, which will be conducted in the future.

In conclusion, we found a novel lncRNA termed RUNDC3A-AS1 that regulated the proliferation and apoptosis of THCA cells via the miR‐151b/SNRPB axis, which may provide a potential biomarker for the research strategy of THCA treatment.

## Data Availability

The datasets used during the current study are available from the corresponding author on reasonable request.
